# Comment on “First-Line *Helicobacter pylori* Eradication with Vonoprazan, Clarithromycin, and Metronidazole in Patients Allergic to Penicillin”

**DOI:** 10.1155/2018/5173904

**Published:** 2018-02-20

**Authors:** Negin Kashani, Amin Talebi Bezmin Abadi

**Affiliations:** Department of Bacteriology, Faculty of Medical Sciences, Tarbiat Modares University, Tehran, Iran

We read with great interest the prospective study by Sue et al. evaluating a 7-day therapy against *H. pylori* using VPZ/CAM/MNZ (VCM) in twenty patients with allergy to penicillin [1]. This survey showed that the effectiveness of VPZ-based regimen is much more than PPI-based therapy applied to those 29 Japanese patients (95% CI: 86.1–100%). At least in Japan, vonoprazan is bringing a novel era in the management of gastric acid-related diseases [2]. Although the study by Sue et al. was a novel prospective trial, there are some ethical and technical issues in light of population limitations which may influence the final conclusion by the authors.

## 1. Generalization of the Finding

The main limitation of this survey is the lack of blinding of the study design. This problem directly reduces the ability of generalization for Japanese patients. As an internal control, they should have used a crossover modification in their design for subjects. This item is highly suggested for similar studies in the future. On the other hand, vonoprazan application is restricted to Japan and there is no clue what would happen if it is prescribed by American, Iranian, or Indian gastroenterologists for *H. pylori*-infected patients! This is the main challenge in treating subjects worldwide.

## 2. Factors Affecting the Success of Treatment

Although both the intention-to-treat and per-protocol effectiveness of VPZ-based eradication were found to be 100% (95% CI: 86.1–100%) in this study, the factors affecting the success rate of *H. pylori* therapy were not checked. For example, the smoking situation and alcohol-drinking habits could be valuable candidates to investigate. The consistency of the current finding (100% eradication rate for therapy) can be reevaluated in the condition of those defined queries in Japanese subjects.

## 3. Clarithromycin and Metronidazole Resistance

It has been documented that clarithromycin-based triple therapy (1–2 weeks) is a standard for Asian countries [3], but we need to first check local levels of antibiotic resistance. In this study, there is no clue about those necessary data. Broadly defined, anti-*H. pylori* therapy using clarithromycin is bound to be defeated in the case of high clarithromycin resistance (>20%). In Japan, clarithromycin resistance has quickly increased, and the current resistance rate is 27.7% [4, 5]. The importance of the current point is higher if we address that the authors aimed to recommend this regimen as the first-line therapy against *H. pylori* infection [1]. Similar to clarithromycin, the story is even worse for metronidazole. The mean range of resistance to metronidazole in Asian countries is more than 55%, a fact which reduces the chance of success in regimens using this futile antibiotic against *H. pylori* [6]. Sue et al. did not report data on susceptibility profile for their patients. In fact, clinicians need to have this pattern to reduce antibiotic resistance in patients.

## 4. Population Size

As a prospective study, it is not far from the mind to expect a better sample size at least for the vonoprazan-receiving group (*n* = 20). In Table 1, it is clear that most of the studies in the case of vonoprazan included more subjects than Sue et al. [1]. Additionally, this study was performed at a single medical center which again limits the chance of generalization of the results. Therefore, the statistical power of the study is under question.

## 5. Ethical Considerations

Although the authors addressed that this study is not a randomized clinical trial, there are some ethical considerations which are already neglected. The authors performed endoscopy surgery for diagnostic purposes using the antral biopsies in bacterial culture. Interestingly, IgG serum analysis was performed which is enough. Now, the query is about the accuracy of ethical consideration. It is definitely unacceptable to use an invasive method in *H. pylori* diagnosis while a noninvasive method is conducted at the same time. Taking together, vonoprazan-based triple therapy showed superiority in treating the *H. pylori* infection in comparison with PPI-based therapy. Meanwhile, we should not forget that an optimal anti-*H. pylori* therapy is what eradicates the bacterium in all subjects with minimal side effects, cost, and ethical considerations.

## Figures and Tables

**Table 1:** Distribution of included patients in vonoprazan-based therapy of *H. pylori* in recent years.

Title	Year	Authors	Number of patients
“Vonoprazan, A Novel Potassium-Competitive Acid Blocker, as a Component of First-Line and Second-Line Triple Therapy for *Helicobacter pylori* Eradication: A Phase III, Randomised, Double-Blind Study”	2016	Murakami et al.	650
“Randomised Clinical Trial: Safety, Tolerability, Pharmacokinetics and Pharmacodynamics of Repeated Doses of TAK-438 (Vonoprazan), a Novel Potassium-Competitive Acid Blocker, in Healthy Male Subjects”	2015	Jenkins et al.	60
“Safety, Tolerability, Pharmacokinetics, and Pharmacodynamics of Single Rising TAK-438 (Vonoprazan) Doses in Healthy Male Japanese/Non-Japanese Subjects”	2015	Sakurai et al.	84
“Vonoprazan versus Conventional Proton Pump Inhibitor-Based Triple Therapy as First-Line Treatment against *Helicobacter pylori*: A Multicenter Retrospective Study in Clinical Practice”	2016	Shichijo et al.	2715
“Safety and Sfficacy of Vonoprazan-Based Triple Therapy against *Helicobacter pylori* Infection: A Single-Center Experience with 1118 Patients”	2016	Nakao et al.	1172
“*Helicobacter pylori* Eradication with Proton Pump Inhibitors or Potassium-Competitive Acid Blockers: The Effect of Clarithromycin Resistance”	2016	Matsumoto et al.	125
“A Novel Potassium-Competitive Acid Blocker Improves the Efficacy of Clarithromycin-Containing 7-Day Triple Therapy against *Helicobacter pylori*”	2016	Noda et al.	146
“Usefulness of Vonoprazan, a Potassium Ion-Competitive Acid Blocker, for Primary Eradication of *Helicobacter pylori*”	2016	Yamada et al.	2507
“Vonoprazan Improves the Afficacy of *Helicobacter pylori* Eradication Therapy with a Regimen Consisting of Clarithromycin and Metronidazole in Patients Allergic to Penicillin”	2017	Ono et al.	88
